# The influence of mechanical bowel preparation in elective colorectal surgery for diverticulitis

**DOI:** 10.1007/s10151-012-0852-3

**Published:** 2012-06-16

**Authors:** H. P. van’t Sant, J. C. Slieker, W. C. J. Hop, W. F. Weidema, J. F. Lange, J. Vermeulen, C. M. E. Contant

**Affiliations:** 1 Department of Surgery, Ikazia Hospital, Montessoriweg 1, 3083 AN Rotterdam, The Netherlands; 2 Department of Surgery, Erasmus University Medical Centre, Rotterdam, The Netherlands; 3 Department of Biostatistics, Erasmus University Medical Centre, Rotterdam, The Netherlands; 4 Department of Surgery, Maasstad Hospital, Rotterdam, The Netherlands

**Keywords:** Colonic diverticulitis, Mechanical bowel preparation, Anastomotic leak, Surgical site infection

## Abstract

**Background:**

Mechanical bowel preparation (MBP) has been shown to have no influence on the incidence of anastomotic leakage in overall colorectal surgery. The role of MBP in elective surgery in combination with an inflammatory component such as diverticulitis is yet unclear. This study evaluates the effects of MBP on anastomotic leakage and other septic complications in 190 patients who underwent elective surgery for colonic diverticulitis.

**Methods:**

A subgroup analysis was performed in a prior multicenter (13 hospitals) randomized trial comparing clinical outcome of MBP versus no MBP in elective colorectal surgery. Primary endpoint was the occurrence of anastomotic leakage in patients operated on for diverticulitis, and secondary endpoints were septic complications and mortality.

**Results:**

Out of a total of 1,354 patients, 190 underwent elective colorectal surgery (resection with primary anastomosis) for (recurrent or stenotic) diverticulitis. One hundred and three patients underwent MBP prior to surgery and 87 did not. Anastomotic leakage occurred in 7.8 % of patients treated with MBP and in 5.7 % of patients not treated with MBP (*p* = 0.79). There were no significant differences between the groups in septic complications and mortality.

**Conclusion:**

Mechanical bowel preparation has no influence on the incidence of anastomotic leakage, or other septic complications, and may be safely omitted in case of elective colorectal surgery for diverticulitis.

## Introduction

In the last decade, evidence challenging the general use of mechanical bowel preparation (MBP) prior to elective colorectal surgery has been reported in the literature. A recent meta-analysis of 14 randomized clinical trials suggests MBP can be safely omitted prior to elective colorectal surgery [1]. However, most of these randomized trials include data covering different types of colorectal surgery (right-sided colectomies, left-sided colectomies and low-anterior resections), and distinction between elective surgery for cancer and inflammatory bowel disease is lacking.

To date, four trials focus on rectal surgery and low anastomosis including two subgroup analyses [2, 3], one case–control study [4] and one randomized trial [5]. Results of these studies showed no difference in anastomotic leakage rates in patients treated with or without MBP. Only the French Research Group of Rectal Cancer Surgery (GRECCAR) demonstrated that rectal cancer surgery without MBP is associated with a higher surgical site infection rate although anastomotic leakage rates were not higher [5]. In contrast, Bucher et al. showed that elective left-sided colorectal surgery was safe without MBP [6]. Besides, patients who did not undergo MBP prior to surgery had a lower postoperative morbidity rate.

Due to controversy between studies concerning the use of MBP mentioned above with heterogeneous indications, surgeons still hesitate to omit MBP in some specific cases of colorectal surgery. This is also the case for patients with recurrent diverticulitis. To date, there is no published data regarding MBP and elective colorectal surgery with an inflammatory component such as diverticulitis.

The prevalence of diverticulosis is estimated at 50–70 % in individuals older than 80 years of age. Diverticulosis is most notable in the left-sided colon with up to 99 % involvement of the sigmoid [7]. Diverticulitis is the most common complication of diverticulosis and affects 15–20 % of patients [8]. The benefit of elective surgery for the prevention of recurrent or complicated episodes of diverticulitis is still a matter of debate [9]. The supposed benefit of preventive resection must be weighed against the possible complications related to surgery, such as anastomotic leakage. Elective surgery for diverticular disease is associated with major complications such as anastomotic leakage in 5–10 % of patients and even with mortality (0–1 %) [10]. However, in patients presenting with persistent complaints and prolonged abdominal tenderness due to diverticulitis affecting their quality of life, an elective resection may be legitimate. Due to the fact that anastomotic leakage occurs more frequently in left-sided resections (most common site for diverticulitis), and because of the presence of an inflammatory component, patients surgically treated for diverticulitis may be prone to anastomotic leakage and other septic complications. Therefore, most colorectal surgeons consider a no MBP regimen in elective surgery for diverticulitis an additive risk factor for postoperative morbidity. In this study, we performed an explorative subgroup analysis of data from a prospective randomized trial to assess the influence of MBP on anastomotic leakage rates and other septic complications in patients who underwent surgical treatment of diverticulitis.

## Materials and methods

This study is a subgroup analysis of a prior large multicenter randomized clinical trial performed by Contant et al. to compare elective colorectal resections and primary anastomosis with and without the use of MBP [10]. In the trial, 1,354 patients were randomized to receive mechanical bowel preparation: 2–4 L of polyethylene glycol bowel lavage solution (Klean Prep) in combination with bisacodyl (11 hospitals) or sodium phosphate solution (2 hospitals) prior to elective colorectal surgery. Endpoints were anastomotic leakage and other septic complications. Exclusion criteria were an acute laparotomy, laparoscopic colorectal surgery, contraindications for the use of mechanical bowel preparation, an a priori diverting ileostomy, and age <18 years old. In the present subgroup analysis, 190 (14 %) out of the 1,354 patients, treated in the period from April 1998 to February 2004, were selected for the present study because they had undergone an elective left-sided colon and/or sigmoid resection with primary anastomosis for diverticulitis.

The diagnosis of anastomotic leakage was based on clinical suspicion (prolonged fever, abdominal pain, local or generalized peritonitis, and leucocytosis) and confirmed during contrast radiography (X-ray or computed tomography (CT) scan) or laparotomy. No effort was made to screen for asymptomatic leakage. A distinction was made between major and minor anastomotic leakage, in which major anastomotic leakage required surgical re intervention, whereas minor anastomotic leakages could be treated conservatively or by radiologic intervention. Wound infection was defined as mild in case of erythema or discharge of seroma and as severe in case of discharge of pus, wound necrosis, or wound dehiscence. The follow-up period was defined as the time from the operation until the first outpatient visit after discharge from the hospital, which usually occurred after 2 weeks.

### Surgical technique

Antibiotic prophylaxis was given intravenously to all patients according to the guidelines for the prevention of surgical site infection issued by the department of infectious diseases of each hospital. All resections for diverticular disease were performed by open laparotomy. Anastomoses were fashioned according to surgeon preference. No exact criteria for the creation of a diverting ileostomy were established, and a diverting ileostomy was applied when deemed necessary by the surgeon. Common reasons for applying a diverting ileostomy were difficult operation, fecal contamination, tension on the anastomosis, very low anastomosis, high number of comorbidities, severe inflammation and incomplete donuts when a circular stapler was used.

### Statistical analysis

Groups were compared with respect to complication rates using the chi-square test or Fisher’s exact test. The same test was used to compare risk groups for anastomotic dehiscence. Comparison of continuous or graded outcomes was determined by the Mann–Whitney test. Multiple regression analysis was performed to evaluate various risk factors simultaneously regarding anastomosis-related failure rates. A *p* value ≤0.05 (two-sided) was considered statistically significant.

## Results

One hundred and three patients received MBP (MBP+) and 87 patients did not (MBP−). A diverting ileostomy was fashioned in 5 MBP+ patients (4.9 %) and in 9 (10.3 %) MBP− patients. Reasons for ileostomy creation were doubt about the integrity of the donuts after stapled anastomosis (*n* = 3), a technically difficult operation (*n* = 5), fecal spillage (*n* = 2), and standard procedure of the surgeon on call (*n* = 4). None of the MBP+ patients received a diverting ileostomy because of inadequate bowel preparation. Nevertheless, there was a trend for MBP− patients to receive a diverting ileostomy more frequently (*p* = 0.08, Table 1).

**Table 1:** Baseline characteristics of patients operated on for diverticulitis (%)

	MBP+ (*n* = 103)	MBP− (*n* = 87)	*p* value
Gender			
Female	55 (53 %)	49 (56 %)	0.107
Male	48 (47 %)	38 (44 %)	
Age (years)			
<60	46 (45 %)	39 (45 %)	0.549
≥60	57 (55 %)	48 (55 %)	
ASA			
I/II	95 (92 %)	79 (91 %)	0.193
III/IV	8 (8 %)	8 (9 %)	
Diabetes			
+	9 (9 %)	3 (3 %)	0.115
–	94 (91 %)	84 (97 %)	
Corticosteroids			
+	3 (3 %)	4 (5 %)	0.248
–	100 (97 %)	83 (95 %)	
Coronary artery disease			
+	15 (15 %)	7 (8 %)	0.120
–	88 (85 %)	80 (92 %)	
Peripheral arterial disease			
+	6 (6 %)	4 (5 %)	0.483
–	97 (94 %)	83 (95 %)	
Smoking			
+	45 (44 %)	25 (29 %)	0.029
–	58 (56 %)	62 (71 %)	
BMI (kg/m^2^)			
≤25	42 (41 %)	40 (46 %)	0.283
>25	61 (59 %)	47 (54 %)	
Diverting ileostomy			
+	5 (5 %)	9 (10 %)	0.08
–	98 (95 %)	78 (90 %)	
Surgeon			
<10 years	77 (75 %)	53 (61 %)	0.023
≥10 years	25 (25 %)	34 (39 %)	
Suture of anastomosis			
Stapled	30 (29 %)	31 (36 %)	0.074
Hand-sewn	72 (71 %)	54 (64 %)	
Type of anastomosis			
End-to-end	67 (66 %)	57 (67 %)	0.129
Side-to-end	26 (26 %)	24 (28 %)	
Other	8 (8 %)	4 (5 %)	
Level of anastomosis			
Colocolic	54 (53 %)	45 (52 %)	0.116
Colorectal	48 (47 %)	41 (48 %)	
Perioperative PC			
≤2	95 (92 %)	85 (98 %)	0.099
>2	7 (8 %)	2 (2 %)	
Operating time (min)			
<120	44 (43 %)	41 (47 %)	0.322
≥120	59 (57 %)	46 (53 %)	
Blood loss (cc)			
≤350	57 (56 %)	43 (50 %)	0.230
>350	45 (44 %)	44 (50 %)	
Contamination			
Minor/moderate	96 (93 %)	81 (93 %)	0.225
Severe	7 (7 %)	6 (7 %)	

*MBP* mechanical bowel preparation, *ASA* American Society of Anesthesiologists, *BMI* body mass index, *PC* packed cells

Anastomotic leakage occurred in 13 (7 %) of the 190 patients. Mechanical bowel preparation was not significantly related to anastomotic leakage: 7.8 % in MBP+ versus 5.7 % in MBP− (difference 2.1, 95 % CI −3.7–5.7 %). Baseline characteristics of patients operated on for diverticulitis are shown in Table 1. More patients of the MBP+ group were smokers but were generally operated on by the more experienced surgeons. The other parameters compared did not differ significantly between the groups.

Table 2 displays the results of univariate analysis of the major risk factors for anastomotic leakage. There was no difference in the listed risk factors for the occurrence of anastomotic leakage between MBP+ and MBP− patients. The same results were obtained when multivariate analysis was performed (Table 3). Septic complications are listed in Table 4. There was no significant difference in septic complication rates or mortality rates between MBP+ and MBP− patients.

**Table 2:** Risk factors for anastomotic leakage in 190 patients who underwent elective surgery for diverticulitis

Risk factor for leakage	*n*/*n* (%)	*p* value (univariate)
MBP		
+	8/103 (7.8 %)	0.79
–	5/87 (5.7 %)	
Gender		
Female	5/99 (4.8 %)	0.35
Male	8/86 (9.3 %)	
Age		
<60 years	4/85 (4.7 %)	0.45
≥60 years	9/105 (8.6 %)	
ASA		
I	3/72 (4.2 %)	0.41
II	8/102 (7.8 %)	
III/IV	2/16 (12.5 %)	
Diabetes		
+	1/12 (8.3 %)	0.58
–	12/178 (6.7 %)	
Corticosteroids		
+	1/7 (14.3 %)	0.40
–	12/183 (6.6 %)	
Coronary ischemic disease		
+	2/22 (9.1 %)	0.65
–	11/168 (6.5 %)	
Peripheral ischemic disease		
+	0/10	1.0
–	13/180 (7.2 %)	
Smoking		
+	2/70 (2.9 %)	0.14
–	11/110 (9.2 %)	
BMI (kg/m^2^)		
≤25	5/83 (6.1 %)	0.72
>25	8/108 (7.4 %)	
Diverting ileostomy		
+	1/14 (7.1 %)	1.0
–	12/176 (6.8 %)	
Surgeon		
Resident	4/82 (4.9 %)	0.64
Surgeon < 10 years	4/48 (8.3 %)	
Surgeon ≥ 10 years	5/59 (8.5 %)	
Suture of anastomosis		
Stapled	2/61 (3.3 %)	0.23
Hand-sewn	11/126 (8.7 %)	
Type of anastomosis		
End-to-end	9/124 (7.3 %)	0.74
Side-to-end	4/50 (8.0 %)	
Other	0/12	
Level of anastomosis		
Colocolic	5/99 (5.1 %)	0.44
Colorectal	8/89 (9.0 %)	
Peri-operative PC		
≤2	11/180 (6.1 %)	0.12
>2	2/9 (22.2 %)	
Operating time (min.)		
<120	4/85 (4.7 %)	0.45
≥120	9/105 (8.6 %	
Blood loss (ml)		
≤350	8/100 (8.0 %)	0.72
>350	5/89 (5.6 %)	
Contamination		
Minor	5/103 (4.9 %)	0.49
Moderate	7/74 (9.5 %)	
Severe	1/13 (7.7 %)	

Due to occasional missing data numbers do not always add up to 190

*MBP* mechanical bowel preparation, *ASA* American Society of Anesthesiologists, *BMI* body mass index, *PC* packed cells

**Table 3:** Multivariate analysis of the listed covariates for their influence on the occurrence of anastomotic leakage

Covariate	*p* value
MBP	0.40
Age	0.68
ASA	0.29
BMI	0.58
Stapled anastomosis	0.59
DM	0.69
Smoking	0.14

*MBP* mechanical bowel preparation, *ASA* American Society of Anesthesiologists, *BMI* body mass index, *DM* diabetes mellitus

**Table 4:** Morbidity and mortality rates after elective surgery for diverticulitis with and without preoperative MBP

Complication	MBP+ (*n* = 103)	MBP− (*n* = 87)	*p* value
Nr of patients with complications^a^	37 (35.9 %)	26 (29.9 %)	0.38
*Anastomotic leakage*			
Minor	2 (1.9 %)	1 (1.1 %)	1.0
Major	6 (5.8 %)	4 (4.6 %)	0.76
*Wound infection*			
Mild	6 (5.8 %)	5 (5.7 %)	0.26
Severe	6 (5.8 %)	1 (1.1 %)	
Urinary tract infection	12 (11.7 %)	7 (8.0 %)	0.41
Pneumonia	9 (8.7 %)	8 (9.2 %)	0.91
Intraabdominal abscess	1 (1.0 %)	4 (4.6 %)	0.18
Fascia dehiscence	5 (4.9 %)	1 (1.1 %)	0.22
Mortality	2 (1.9 %)	2 (2.3 %)	1.0

^a^Patients can have more than one complication at a time

## Discussion

In this study, we aimed to assess the value of preoperative MBP in patients undergoing elective colorectal surgery for diverticulitis (Hinchey I/II). Although our study is a subgroup analysis, it is, to the best of our knowledge, the first study in the literature to focus on the value of MBP before elective colorectal surgery for diverticulitis. We found that elective colorectal surgery without MBP was not significantly associated with a higher anastomotic leakage rate (7.8 vs. 5.7 %, *p* = 0.79) or other septic complications (35.9 vs. 29.9 %, *p* = 0.38). The present study did show a trend toward a higher incidence of intra-abdominal abscesses in the MBP− group, corresponding to the results in the primary multicenter randomized trial from which this subgroup was derived by Contant et al. [11]. However, this difference did not become statistically significant (1.0 vs. 4.6 %, *p* = 0.18).

The prevalence of diverticulosis in Western countries is high and increases with age. A study by Mendeloff et al. reports that one-third of the general population of the United States had developed diverticulosis by the age of 45 years and two-thirds by the age of 80 [12]. Although most patients will remain asymptomatic, 10–20 % will develop symptoms or complications [13]. Traditionally, patients were advised to undergo resection of the affected colon segment after two episodes of diverticulitis due to a supposed higher risk of complications (fistula, abscess formation, and perforation) and even mortality in case of recurrence [14, 15]. At present, the indication and timing for elective surgery for diverticulitis is a matter of debate as elective colon resection is not risk-free. Eglinton et al. and Janes et al. challenge the dogma of surgery after two attacks of diverticulitis and support a more conservative approach. They weigh the morbidity and mortality associated with subsequent episodes of diverticulitis in patients treated conservatively against the morbidity and mortality associated with elective resection. They conclude that elective resection performed after two attacks of diverticulitis to prevent recurrence or the development of complications should not be routine management [12, 16].

Resection with primary anastomosis in patients with diverticular disease is associated with higher rates of morbidity and mortality compared to elective colorectal resection for colon cancer [17]. This is why many colorectal surgeons are reluctant to omit MBP prior to elective surgery for diverticular disease.

In theory, MBP is believed to clean the colon and rectum of remaining feces in order to reduce the bacterial load and protect the patient against postoperative anastomotic and infectious complications [18]. This may well be true for patients undergoing left-sided colectomies when an infectious component such as diverticulitis is involved. However, the effect of MBP prior to surgery for diverticulitis in lowering morbidity and mortality rates has not been thoroughly investigated. Two studies investigated risk factors for anastomotic leakage in sigmoid colectomy for diverticulitis. Lehmann et al. note that stapled anastomosis was associated with lower leak rate than hand-sewn anastomosis, and Levack et al. found that anastomotic leakage occurred less frequently after laparoscopic surgery compared to open surgery for diverticulitis [19, 20]. Neither studies mention the use of MBP. Two other studies investigated primary resection and anastomosis with intraoperative colonic lavage compared to Hartmann’s procedure for complicated diverticulitis with peritonitis. The authors are in favor of primary resection and anastomosis with intraoperative colonic lavage aimed at reducing anastomotic complications [21, 22]. None of the studies mentioned consider whether MBP or colonic lavage should be applied in elective colorectal surgery for Hinchey stage I or II diverticulitis. The present study only included patients with Hinchey stage I and II diverticular disease. Mechanical bowel preparation was not related to the occurrence of anastomotic leakage, other septic complications, or mortality.

The present study has some limitations. As mentioned before, this is a subgroup analysis, and the data used were derived from an earlier multicenter randomized trial designed for a different purpose [11]. About half of the patients underwent sigmoid resection with colo–colonic anastomosis, whereas generally recommended surgical treatment in cases of diverticulitis involves resection with a distal margin at the upper rectum. The risk of recurrent diverticulitis might be lower when resection extends to the proximal rectum [23], and this seems to be correlated with a lower risk of anastomotic leakage. The existing literature about this issue is rather limited and not uniform.

In addition, no distinction was made between Hinchey stage I and II diverticular disease. Data such as type and duration of complaints, number of episodes of diverticulitis and prior antibiotic treatment (besides antibiotic prophylaxis), which may be related to the outcome of surgery, were not collected. A recent meta-analysis has shown a significant decrease in wound infection complications after surgery in patients receiving oral antibiotics with MBP compared with intravenous antibiotic prophylaxis [24]. In this study, the patients received routine intravenous antibiotic prophylaxis. The risk of anastomotic leakage in patients receiving intravenous antibiotics alone was not increased [24], but recently, a prospective randomized trial has started to investigate this issue further.

## Conclusions

Mechanical bowel preparation before elective colorectal surgery for diverticulitis, Hinchey stage I and II, is not related to the occurrence of anastomotic leakage and other septic complications. It therefore appears that MBP could safely be omitted for patients scheduled to undergo elective resectional surgery. However, this statement is based on a subgroup analysis of an earlier multicenter randomized trial designed for a different purpose. Therefore, more prospective randomized, designed studies are warranted.
